# An investigation of secondary electron emission from ZnO based nanomaterials for future applications in radiation detectors

**DOI:** 10.1038/s41598-020-80788-y

**Published:** 2021-01-12

**Authors:** P. Boutachkov, K. O. Voss, K. Lee, M. S. Song, C. Yi, M. Cappellazzo, W. Kondziołka, A. Liskowicz, M. Cholewa

**Affiliations:** 1 GSI Helmholtzzentrum Für Schwerionenforschung GmbH, Planckstraße 1, 64291 Darmstadt, Germany; 2 Department of Physics and Astronomy, Institute of Applied Physics and Research Institute of Advanced Materials (RIAM), Seoul National University, Seoul, 08826 Republic of Korea; 3 Institut Für Kernphysik, University of Cologne, Zülpicher Straße 77, 50937 Köln, Germany; 4 Institute of Physics, College of Natural Sciences, University of Rzeszów, Pigonia Street 1, 35-959 Rzeszów, Poland

**Keywords:** Nanoscience and technology, Physics

## Abstract

This paper discusses the use of nanomaterials for the improved performance of time-of-flight particle detectors based on secondary electron emission (SEE). The purpose of the research presented in this paper is to find a nanomaterial that has a higher SEE than gold. In this article, we present a measurement of the SEE properties from 1D (one-dimensional) nanostructures of ZnO and ZnO/GaN (ZnO with GaN coating) composed of a mostly regular pattern of nanotubes grown on a thin Si_3_N_4_ substrate. The study was performed with 4.77 meV/u Au beam. We observed an average increase of 2.5 in the SEE properties from the 1D ZnO nanotubes compared to gold.

## Introduction

Stimulated electrons in the interaction of charged particles and radiation with matter which escape the material surface are called secondary electrons^1^. This secondary electron emission (SEE) is utilized in particle and radiation detectors^2,3^. The secondary electrons stimulated near the material surface have a better chance to escape the material, therefore nanomaterials may improve the SEE yield through the manipulation of material geometry. Further modification of the surface coating can be used to decrease the energy necessary for the electrons to leave the materials surface, further enhancing the SEE yield.

Significant work has been performed on the enhancement of SEE properties under different conditions for various applications, centered towards applications in flat screens^4^, the detection of electrons, and X-rays^3^. The 1D nanotubes were investigated by Cholewa et al.^5^. These results motivated the present study, wherein using nanomaterials in thin SEE detector for heavy ions was investigated. A typical detector of this type will utilize thin gold or carbon foil(s). The ions of interest will pass through the foil inducing SEE. The electrons will be collected with electrical fields and subsequently detected. An example of such a detector can be found in^5^.

In this work, we present the data from ZnO nanotubes and GaN coated ZnO nanotubes. ZnO nanotubes with a diameter of 1 µm and a length of 5 µm were employed for this research and the GaN was covered with 10 nm on the ZnO nanotubes^6^. Additionally, a 20 nm Au deposited on thin (1 µm) silicon nitride (Si_3_N_4_) substrate sample was used as a reference/normalization.

We also used thin (1 µm) Si_3_N_4_ foils as a substrate for ZnO nanotube growth.

## Methods

All samples were prepared on a 1 µm thick Si_3_N_4_ windows from SILSON Ltd in the UK. To enhance the electric conductivity, for a more homogeneous electric field of the applied bias voltage, and for growing the nanostructures, chemical vapor deposited (CVD) graphene films were transferred on a Si_3_N_4_ substrate. Subsequently, position-controlled ZnO structures were grown via Metal Organic Vapor Phase Epitaxy (MOVPE), as described by Park et al.^6^. Following this, the GaN was coated on the ZnO nanotube arrays.

## Sample preparation

Preparation of the substrate for the selective growth of ZnO nanotubes consisted of the deposition of a 50 nm of SiO_2_ growth mask on the CVD graphene films using the PECVD (Plasma-Enhanced Chemical Vapor Deposition) system. The oxide layer was annealed at 600° C in an oxygen rich condition to reduce the oxygen defect in the deposited SiO_2_ growth mask, which could lead to undesirable growth. The next step was to determine the pattern of the sample traces using electron beam lithography. The patterned SiO_2_ film was then dry etched with CF_4_ plasma, and the remains of the oxide layer on graphene films after the dry etching process was removed with buffered oxide etchant (BOE). Before any growth, samples were cleaned with acetone, isopropyl alcohol and nitric acid.

**Figure 1:**
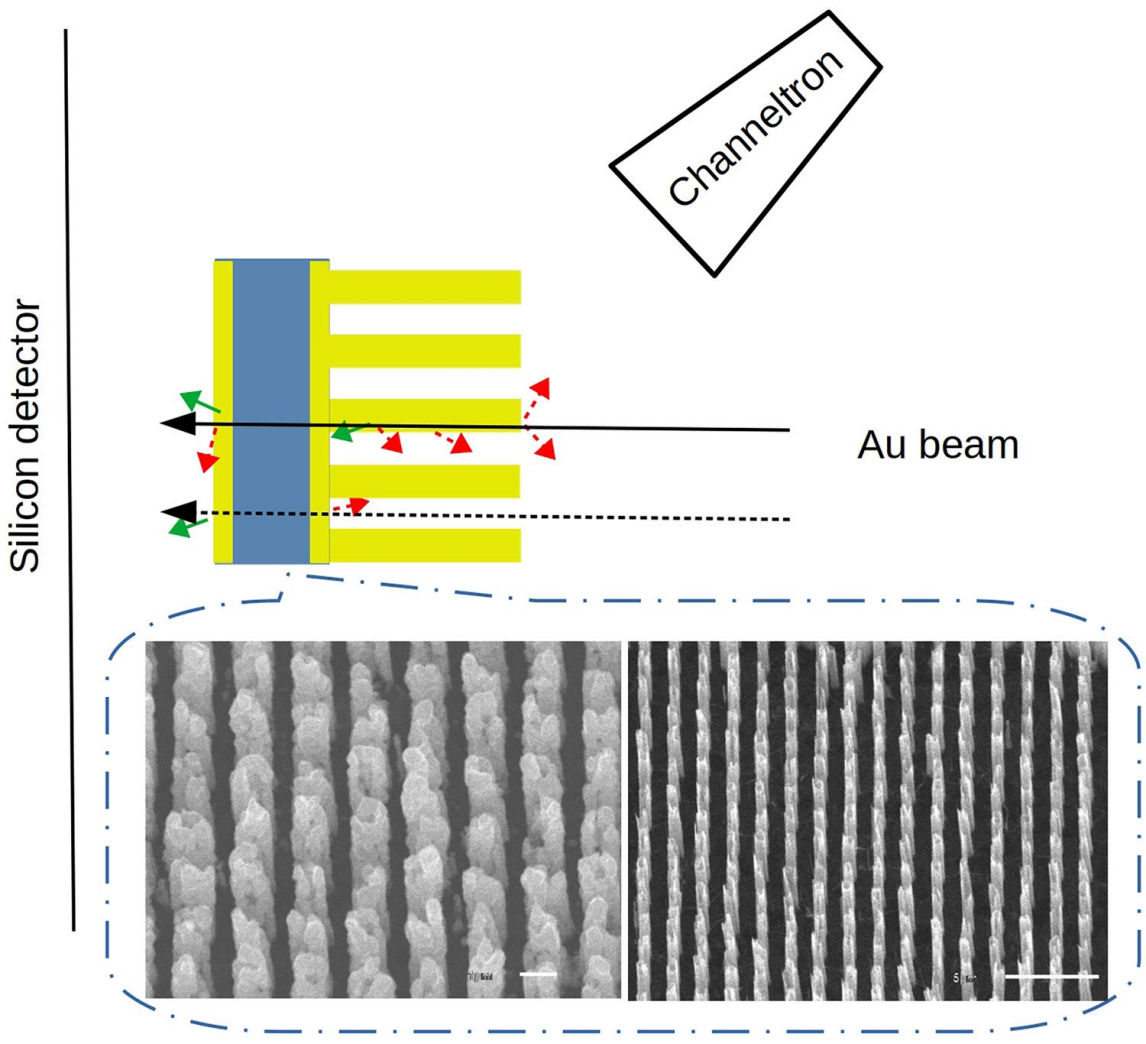
A schematic of the experiment setup (not to scale). The insert shows SEM image**s** of the investigated nanostructures: ZnO/GaN (left) with a scale of 1 µm and ZnO (right) with a scale of 5 µm. GaN coating was 10 nm thick and covered uniformly the top and side of nanorods and bottom of the Si_3_N_4_ film supporting the nanostructures.

After the the ZnO nanotube growth (Fig. 1), they were coated with a GaN about 10 nm thick using low-pressure metal–organic phase epitaxy (MOVPE) using trimethyl-Ga (TMGa) as a precursor^7^. The GaN layer was deposited at both ends of ZnO nanotubes, and evenly coated along the side wall^6,8^.

The nanotube arrays were examined by scanning electron microscopy (SEM) and transmission electron microscopy (TEM) after production. And they presented uniform morphologies and high structural quality over a large area and could be prepared on a broad variety of substrates, including amorphous, metallic, flexible substrates and thin Si_3_N_4_ commercial films^6^.

Bottom-up growth of one-dimensional (1D) semiconductor nanostructures has been extensively studied for fabricating different nanodevices including sensors, light-emitting diodes, transistors, and photovoltaic cells^9–11^.

The proposed coating is based on the wide bandgap (Eg = 3.4 eV) semiconductor gallium nitride (GaN). The electrical conductivity of GaN can be controlled over a wide range through modifications to the film composition or structure. GaN is also refractory and chemically stable. Such material had been studied before^8^ with carbon ions and we observed higher SEE properties when compared with uncoated ZnO nanomaterial.

## Experiment

All measurements were performed at GSI Helmholtzzentrum für Schwerionenforschung GmbH, Germany. A 4.77 meV/u gold beam was delivered by the UNILAC^5,12,13^ accelerator to the microbeam experiment setup. Details of the system forming a heavy ion microbeam in GSI is described by Fischer et al.^5^. The Au beam was focused to a ca. 0.1 mm beam spot. The heavy ions had sufficient energy, effectively penetrating the samples. The ions were stopped in a silicon surface barrier detector. A BURLE Electro-Optics channeltron Model 4028 was position 5 mm upstream from the target and a 180 V potential difference was applied between the sample surface and the channeltron, guiding the SEE electrons to the detector. Schematics of this experiment setup are shown in Fig. 1.

In order to assess the efficiency of the system, and to confirm there were no unknown sources of electrons, data was taken with the acquisition system triggering on the silicon detector, and then the trigger was changed to the channeltron signal. The shaped signal from the channeltron and the silicon detector were simultaneously recorded. The average beam intensity was less than 50 particle/s, leading to no acquisition dead-time.

When triggering the Si detector, the number of events where the Si detector had a valid signal, above noise, was equal to within 0.02% to the number of events from the channeltron. Therefore, all Au ions which passed the targets created secondary electrons, part of which were detected by the channeltron.

The Au ions pass through the investigated sample and halt in a Silicon detector. The channeltron registers secondary electrons collected from the surface of the target. The secondary electrons are schematically depicted with red and green arrows. The green arrows symbolize δ-electrons, which can create further secondary electrons if they encounter the sample surface In the inset, SEM pictures of the investigated nanostructures, to the left ZnO covered with GaN, and ZnO without coating to the right. The nanotubes, with spacing and diameters, are: 1.57 µm and 0.6–0.7 µm for the sample shown to the left; and 1.57 µm and 1–1.3 µm for the sample shown to the right. The approximate nanorod length is 5 µm, which is perpendicular to the surface.

**Figure 2:**
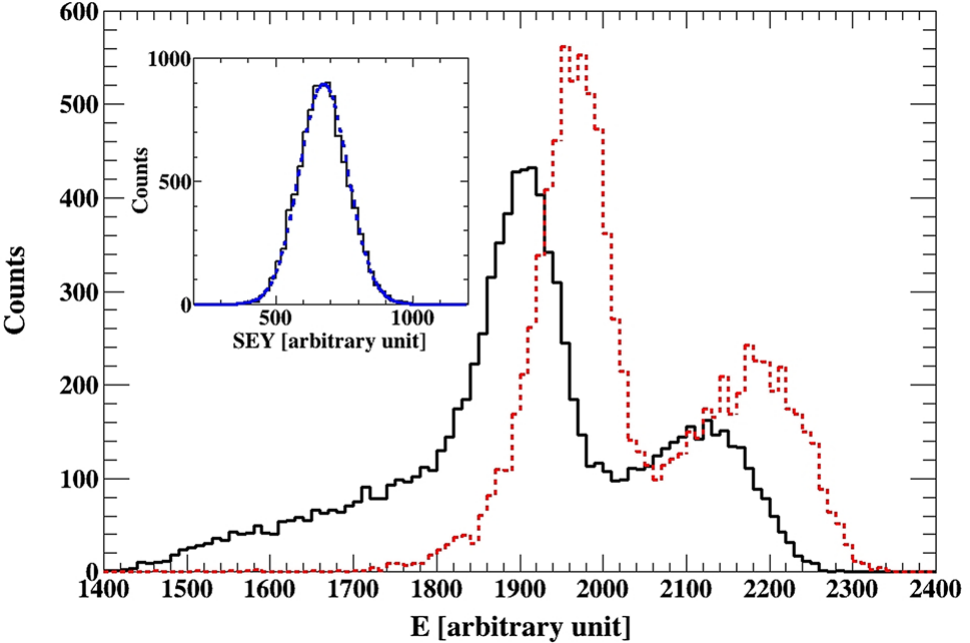
Energy spectrum measured with the Si detector for Au and ZnO**/**GaN samples. The ZnO**/**GaN data is shown with the black unbroken line. The data from the Au sample is shown with the red dashed line. See text for further details. The inset shows the SEY measured from the Si_3_N_4_ foil covered with 20 nm thick layer of gold. It is fitted with a normal distribution. The peak is well described by the fit pointing to a single source of secondary electrons.

In the data presented below, a gate requiring signal above noise for both silicon and channeltron detectors, was applied. Thus, selecting events where the gold ions pass through the sample, created secondary electrons and interact with the active volume of the Si detector. In order to provide an absolute comparison of the measured secondary electron yield (SEY) to other experiments, we measured the SEY from the gold sample. The SEY spectrum obtained from the Au sample is shown at the inset of Fig. 2. The mean value of the distribution is proportional to the average number of secondary electrons created by a single gold ion passing through the sample. The area under the peak is equal to the number of ions which passed through the sample, while the distribution width is determined by the statistical processes involved in the creation of the SEY and their detection. Therefore, the two parameters of interest are the mean and width of the measured distribution. The data for the sample discussed below are normalized to 10,000 Au ions detected by the Si detector, whilst coinciding with secondary electrons.

The experiment utilizes the difference in energy loss of the Au beam ions when passing through a nanotube or a lull between the tubes. Due to the two distinct energy losses, one can expect two peaks in the Si detector amplitude spectra, a low energy peak corresponding to the ions which lose more energy when they pass through the nanorods and a higher energy peak, corresponding to the ions which pass in the space between the nanorods. Figure 2 shows the spectrum registered by the Si detector for an Au sample and a ZnO/GaN sample. The gold sample is homogeneous, yet there are two peaks in the measured spectra. This is related to a damage of the surface barrier detector. The detector is mechanically moved during data collection. This movement is introduced in order to minimize the risk of damaging a particular area of the detector. If the response of the detector is different along its surface, we might observe a structure in the measured beam energy, which is the case in Fig. 2. In a latter study, we compare the response of the detector in question, and a brand new detector. The double hump/peak was not present in the spectrum measured with the new detector.

## Results

When comparing the shape of the two spectra shown in Fig. 2, one can see that the continuous black spectra for the ZnO sample is shifted to the left relative to the spectrum from the Au sample. This is due to the higher energy loss in the ZnO sample. Furthermore, there is a tail to the left corresponding to lower energies. These events should correspond to the Au ions which pass through the ZnO nanotubes, and thus lose more energy.

**Figure 3:**
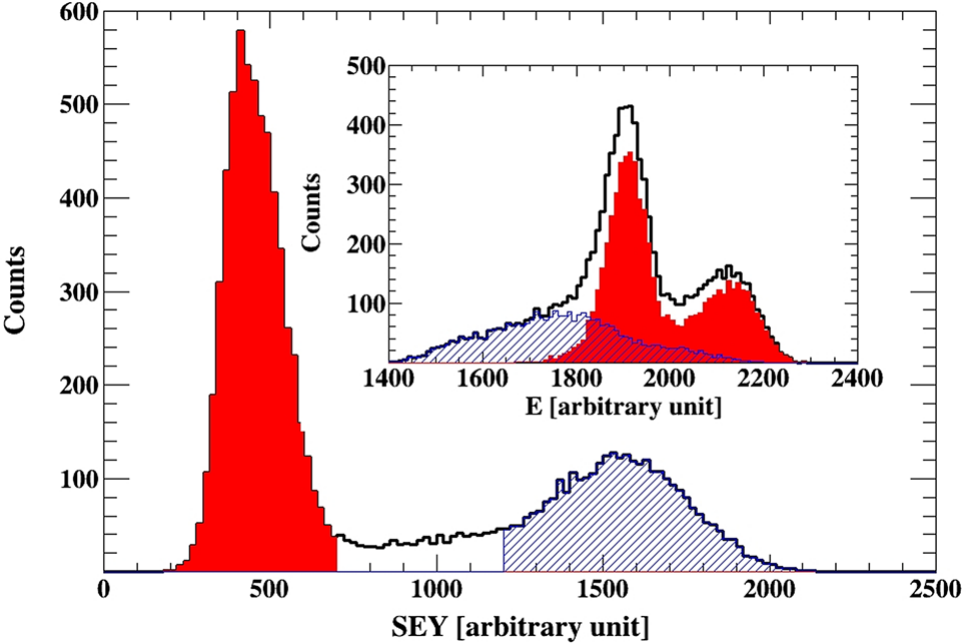
The SEY measured for the ZnO/GaN, where the inset shows the corresponding spectrum obtained from the Si detector. Gates are placed on the SEY data, the corresponding events in the Si detector are shown in the inset. The result demonstrates the relation between higher SEY yield and lower energy in the Si detector, and lower SEY yield and higher energy deposited by the Au ions in the silicon detector. The higher SEY yield is related to Au ions passing through the nanotube, while the lower yield is observed for ions passing in the lulls/drops between the nanotubes.

**Figure 4:**
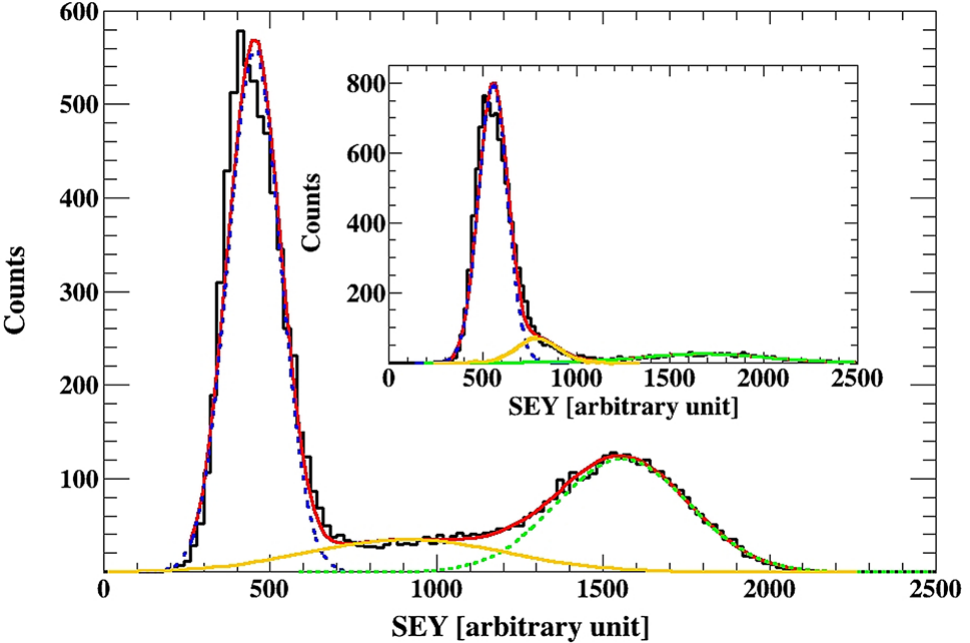
The SEY for the ZnO/GaN sample fitted with 3 Gaussians. The inset show the data for the ZnO sample (without GaN coating). The ratio of the area under the high SEY distribution and the total number of counts in the SEY spectra is 0.3 for the ZnO/GaN sample, and 0.1 for the ZnO sample. These are consistent with the geometrically calculated ration of 0.3–0.5 and 0.11–0.16, respectively.

The inset of Fig. 3 show the spectra observed from the Si detector for the ZnO/GaN, and the corresponding SEY spectra observed in the channeltron. There are two dominant peaks in the secondary electron spectra. If we gate on the higher peak in the SEY spectra, the blue dashed area, we select the events shown with the same pattern in the Si spectra. Gating on the lower SEY peak, shown in solid red, selects events which have a higher energy, as indicated in the corresponding Si spectra. Therefore, based on the above results and contentions, it can be concluded that the measured higher electron yield is related to Au ions, which have lower energy after the ZnO samples, while the lower SEY peak occurs due to the lulls between the nanotubes, giving higher signal in the Si detector. For a comparison of the SEY spectrum from the Au sample, shown in the inset of Fig. 2, shows one peak, as expected, from a homogeneous material (Fig. 4).

## Discussion

The measured SEY spectra can be fitted with a sum of 3 normal distributions. In Fig. 3 the fit for the corresponding spectra obtained from ZnO/GaN and the ZnO (without GaN coating) are shown. The same samples are shown in the inset of Fig. 1. The higher SEY peak can be well described by a normal distribution. While for the low SEY yield, the peak is not well described around the peak maxima. Hence, there is a single source with higher secondary yield related to the nanotubes. There are at least 3 sources of lower secondary electron yield—two of these are represented by the two Gaussian distributions used in the fit. The presence of a third source is indicated by the poor fit for the low SEY peak.

The area under the SEY peaks should be related to the area presented to the beam from the different sources. The counts under the higher SEY peak to the total number of counts in the SEY spectra, is expected to be equal to (the area of the nanotube exposed to the beam)/(arc of the valleys) = π (nanotube radius)^2^/(distance between the nanotubes)^2^. The measured and calculated rations are 0.3 and 0.3–0.5 for ZnO/GaN and 0.1 and 0.11–0.16 for ZnO, respectively. This supports the interpretation that the higher SEY peak is related to Au ions interacting with the ZnO nanotubes.

The calculated energy loss of Au ions in the sample, further confirms the above analysis. There is an uncertainty in the ZnO average density that should be used in the calculation, we assumed 5.5 g/cm^3^. A calculation with ATIMA^14^ yield:

E(after passing through a nanotube)/E(after passing through a lull) = (3.5 meV/u) / (4.6 meV/u) . Assuming 10% error in the energy loss calculation a ratio of 0.76(11) is obtained. Hence, a peak in the Si spectra in channel 2.1 k, due to an ion passing through a lull, should have a corresponding peak in channel 1.6(3) k. The second peak, due to the defect Si detector, centered around 1.9 k will have a corresponding peak centered around 1.4(3) k due to ions passing through nanotubes. Considering the uncertainties in the energy calculation this result is consistent with the data shown in the inset of Fig. 3.

**Figure 5:**
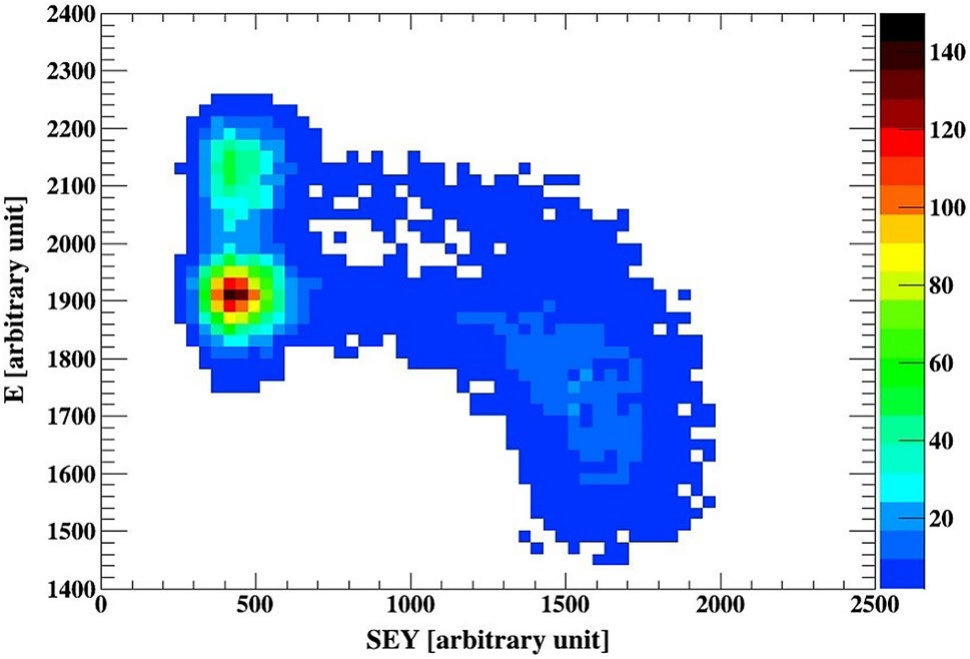
The energy measured with the Si detector versus SEY for the ZnO/GaN sample. The histogram binning for both observables is reduced by a factor of 2 compere to the rest of the histograms show in this work. The histogram demonstrates the correlation between the energy loss in the sample and the SEY. Higher SEY is related to Au ions with lower energy after the sample, i.e. ions which passed through a nanotube. The continuous change of SEY between channels 600 and 1200 as a function of energy after the target can be caused by ZnO material deposited in the lulls or broken tubes.

The relations between the energy deposited in the Si detector and the SEY discussed above are reflected in the two dimensional histogram shown in Fig. 5. An additional feature is visible around channel 1400 of the SEY. The yield reduces as the deposited energy in the Si detector increases. There is insufficient data to explain this relation. A possible clue is visible in the SEM image shown in Fig. 1, the nanotubes are not perfect cylinders perpendicular to the substrate. The large energy spread measured for ions passing through a nanotubes compere to the one that went through a lull indicates a variation of the amount of ZnO material in the ion path. The SEM image shown in the inset of Fig. 1 shows the irregular structure of the nanotubes. Calculating the energy loss variation due to nanotube length change by 1 µm leads to a shift of order of 100 channels in the energy spectra shown in Fig. 5.

The results of the measurements are summarized in Table 1. An average increase of the SEY from ZnO nanotubes of 2.5 times compared to the gold sample, was observed. The data shows that GaN coating does not lead to a higher SEY yield.

**Table 1:** Data on the SEY yield denoted with δ for the samples discussed in this paper.

Sample	δ_1_/δ_1_^Au^	σ_1_/δ_1_	δ_2_/δ_1_^Au^	σ_2_/δ_2_	δ_3_/δ_1_^Au^	σ_3_/δ_3_
Au	1	0.13	–	–	–	–
ZnO/GaN	0.7	0.17	1.3	0.3	2.3	0.13
ZnO	0.8	0.14	1.2	0.15	2.5	0.2

σ is indicative of the standard deviation determined from the fits shown in Fig. 4.

## Outlook

What is interesting in the presented data, where we used Au heavy ion beam probe, we did not find any increase in the SEY due to the GaN coating. This is an open question for further investigation.

From this research we have several conclusions: (1) the increase in the SEY from the investigate nanomaterial is of order of 2.5 when compared to Au foil (2) the GaN coating did not lead to measureable increase in the SEY compere to non-coated nanomaterial. This is different from observation in ref.^8^ which could be attributed to the fact that the carbon ions were used in the past.

From point of view if practical detector applications, the measured increase in SEY yield comes at a price of beam with broader energy spread after detection.

One possible direction in future investigations is: (1) search for coating leading to larger SEY (2) investigating materials with smaller nano-tube diameter and larger density. These investigations may lead to increase of the SEY.

Here we would like to suggest an alternative direction for future material development and experimental investigations where the SEY can be increased by more than an order of magnitude, while the energy broadening will approach the energy straggling introduced by a new µm of homogeneous ZnO layer. This idea is introduced bellow.

**Figure 6:**
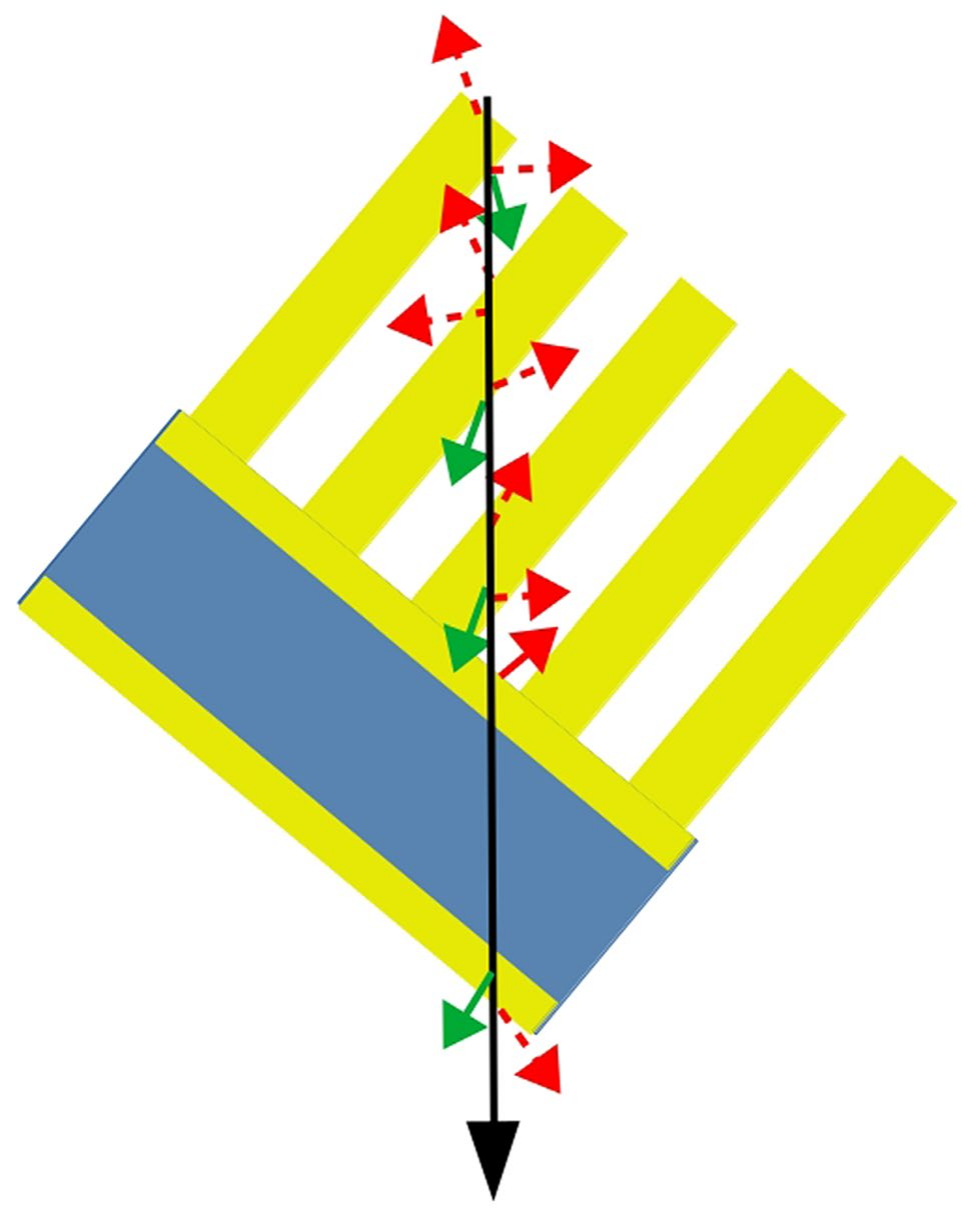
A concept of using a nanostructure to increase the SEY per ion. a) a side view of a regular nanostructure. The heavy ions depicted by the black arrow/line interact with the sample knocking slow electrons depicted in red, and δ-electrons depicted in green. A significant gain in the SEY is obtained due to passing through multiple entrance and exit surfaces.

When considering thin detectors we suggest the use of a nanomaterial made by growing high aspect ridges on a thin substrate, with ridges regularly spaced and perpendicular to the substrate. If this material is placed at an angle relative to the beam, as illustrated in Fig. 6, the ions will pass through multiple ridges. Utilizing this approach, the SEY is increased due to passing through multiple surfaces. It is worth noting that presenting further surfaces due to the to the higher energy δ-electrons bumped by a neighboring nanostructure, the SEY will be further increased via secondary interactions with the material. These δ-electrons are schematically depicted as green arrows in Fig. 6.

In the sketched example the ion trajectory passes through 8 surfaces, compare to 2 for a flat foil. Hence the SEY yield will increase by more than 8*2.5/2 times compare to foil covered with gold. Here we assume that all secondary electrons can be extracted from the volume between the ridges vial electrical field. The exact gain will be determined from the geometry of the nanoridges, e.g. high, spacing, width and the materials used to build them.

In the above approach, the energy spread is significantly reduced as the variation of the total geometric length of the ion track in the nanomaterial is reduced to (ridge thickness)/cos(angle between the beam and the substrate surface). For example, a ridge structure placed at 45° relative to the beam, with 0.5 µm thick ridges, ridge depth of 2.5 µm placed 0.5 µm apart will introduce energy spread of the beam equivalent to of 0.7 µm of ZnO. Which corresponds to energy broadening of order of 3% for Au ions beam at 4.77 meV/u, while the average ion path through the foil will be 1.75 µm of ZnO + (substrate thickness)/cos 45°.

The above purely geometrical considerations indicate a possibility for a major improvement of TOF detector based on SEE detection.

## Conclusions

We have measured the secondary electron yield from 1D ZnO based nanomaterials with 4.77 meV/u gold ions. We used thin samples, utilizing the different energy loss of the probe ions in said samples, in order to separate the SEY from nanorods and the lulls. This approach allows for the differentiation of the SEY yield from nanostructures and other features without specialized accelerator systems delivering micro or nanobeams. This technique can be used to investigate samples with radiation sources, for instance alpha sources, and in bench-top experiments.

There is no narrow peak in the secondary electron yield for the studied structures. This is related to the larger diameter of the ion beam compared to the diameter of a single nanotube. The observed yields from the ZnO nanorods are, on average, 2.5 times higher compared to gold samples.

We propose a concept for a detector based on a tilted thin foil with nanoridges. Combining the observed increase of the SEY from ZnO nanorods with the proposed detector concept would significantly increase the SEY for the transition of ion detectors compared to a detector with a gold foil. The planned solution addresses the problem at hand with an increased energy spread due to the nanostructures.

## Data Availability

The data that support the findings of this study are mostly available within the paper. Additional data is available upon request from the corresponding author.
